# Exploration of Predictive Biomarkers for Postoperative Recurrence in Chronic Rhinosinusitis with Nasal Polyps Based on Serum Multiple-Cytokine Profiling

**DOI:** 10.1155/2022/1061658

**Published:** 2022-09-28

**Authors:** Gang Wang, Huiyuan Zheng, Xiaoqian Chen, Jing Zheng, Jiabin Zhan, Rui Li, Yanyan Qi, Yi Ye, Min Zeng, Xin Wei

**Affiliations:** ^1^Department of Otorhinolaryngology-Head and Neck Surgery, Hainan General Hospital, Hainan Affiliated Hospital of Hainan Medical University, Haikou, China 570311; ^2^Medical Center, Hainan General Hospital, Hainan Affiliated Hospital of Hainan Medical University, Haikou, China 570311

## Abstract

**Background:**

Functional nasal endoscopic surgery (FESS) is an effective treatment approach for chronic rhinosinusitis with nasal polyps (CRSwNP) patients, but some patients still suffer from postoperative recurrence. This study is aimed at investigating the expression of multiple cytokines in CRSwNP and revealing their relationships with postoperative recurrence.

**Methods:**

A total of 72 patients with CRSwNP, including 36 primary and 36 recurrent patients, were enrolled. Serum samples were obtained, 30 cytokine levels were measured by multiplex analysis, and the association between cytokine levels and recurrence was assessed. The most potential cytokines were further validated in another independent cohort with 60 primary and 60 recurrent CRSwNP patients.

**Results:**

The results of multiple cytokine profiling exhibited that the levels of eotaxin, G-CSF, IFN-*α*, IL-13, IL-17A, IL-5, MCP-1, and RANTES were vastly changed in the recurrent group in comparison with the primary group. Receiver-operating characteristic (ROC) curves highlighted that serum levels of eotaxin, IL-17A, and RANTES were strongly predictive of postoperative recurrence (area under the curve (AUC) > 0.7, *P* < 0.05). Further validation results showed that elevated serum eotaxin, IL-17A, and RANTES levels were enhanced in the recurrent group. The ROC curve showed that serum eotaxin (AUC = 0.729, *P* < 0.001) and RANTES (AUC = 0.776, *P* < 0.001) exhibited stronger ability than serum IL-17A (AUC = 0.617, *P* = 0.027) in predicting CRSwNP recurrence.

**Conclusion:**

Our data suggested that serum multiple cytokine profiling was associated with postoperative recurrence of CRSwNP, and eotaxin and RANTES might serve as potential biomarkers for predicting postoperative recurrence. These results might contribute to the understanding of the underlying mechanisms of recurrence and provide novel clues for precision therapy in CRSwNP.

## 1. Introduction

Chronic rhinosinusitis (CRS) is a chronic heterogeneous inflammatory disease which has affected about 8% of the Chinese population and 10–15% of the European population [1, 2]. Based on nasal endoscopy and computed tomography (CT) findings, CRS is generally categorized into two phenotypes: CRS with nasal polyps (CRSwNP) and CRS without nasal polyps (CRSsNP) [3, 4]. It is well known that CRSwNP is characterized by the accumulation of inflammatory cells and the release of multiple cytokines in the nasal mucosal tissue, which lead to diverse endotypes [5–8]. Although the clinical symptoms of CRSwNP and nasal inflammation can be alleviated by functional endoscopic sinus surgery (FESS), more than 30% of patients still suffer recurrence 8 years after surgery [9]. Vlaminck et al. [10] reported that the overall recurrence rate of CRSwNP was more than 50% three years after FESS. Considering the high postoperative recurrence rate of CRSwNP, preoperative identification of these postoperative recurrence-prone patients facilitates the treatment selection and improves personalized therapy. Therefore, it is urgently needed to explore objective biomarkers to predict postoperative recurrence of CRSwNP.

Cytokines are mainly produced by activated immune cells, including T cells and B cells, and play essential roles in the development of immune responses. Cytokine level measurement provided a novel clue to discovering underlying pathomechanism in various clinical disorders [11–13]. Previous studies demonstrated that multiple cytokine analyses in serum or other liquid specimens were successfully applied to explore biomarkers for disease diagnosis and prognosis prediction in inflammatory and allergic diseases [14]. Zhang et al. [15] demonstrated that serum multiple-cytokine profiling was associated with the response of sublingual immunotherapy in AR patients, and serum IL-10 and IL-33 might serve as novel biomarkers for early efficacy prediction. A recent study found that several cytokine levels were closely associated with adverse effects in gastroschisis, and they could be used as simple indicators to predict the adverse outcomes [16]. However, little is known about the serum cytokine profile in CRSwNP patients, especially in recurrent patients. Thus, investigating factors associated with postoperative recurrence of CRSwNP based on multiple cytokine profiles is of great clinical significance. Therefore, we conducted this discovery and validation study to investigate the expression of multiple cytokines in CRSwNP and determine the predictive values of candidate cytokines in CRSwNP recurrence based on multiple cytokine profiles.

## 2. Methods

### 2.1. Subjects and Setting

A total of 72 CRSwNP patients who underwent FESS between January 2021 and March 2021 at our department were recruited in this study. All patients underwent physical examination, endoscopic findings, or computed tomography (CT) scans in the outpatient department and were diagnosed with CRSwNP according to the criteria recommended in the European Position Paper on Rhinitis and Nasal Polyps [1]. Exclusion criteria for this study included: (1) patients <18 years or >65 years; (2) patients who had received systemic or topical corticosteroids, antibiotics, or other immunomodulatory medications within 4 weeks prior to FESS; (3) patients with allergic fungal rhinitis and nasal or sinuses carcinoma; (4) other systemic inflammatory and eosinophilic diseases. Clinical data were collected, including sex, age, body mass index (BMI), comorbidity, blood eosinophil count and percentage, and visual analogue scale (VAS) value of nasal symptoms. Preoperative CT and nasal endoscopy scores were recorded by the Lund-Mackay and Lund-Kennedy score systems, respectively [17].

### 2.2. Sample Collection and Cytokine Measurement

Five mL of peripheral venous blood samples were obtained from each patient before FESS and stored at room temperature for 1-2 hours. All specimens were centrifuged for 10 min, and the supernatants were collected and stored at -80°C for subsequent experiments. Serum multiple cytokine profiling was performed in the MAGPIX system (Luminex) using a multiplex assay kit (Bio-Rad, CA, USA) referring to the manufacturer's instructions. The commercial kit consists of the following 30 cytokines: cutaneous T cell attracting chemokine (CTACK), eotaxin, granulocyte colony-stimulating factor (G-CSF), granulocyte-macrophage colony-stimulating factor (GM-CSF), interferon alpha (IFN-*α*), IFN-*γ*, interleukin (IL)-10, IL-13, IL-15, IL-16, IL-17A, IL-18, IL-1*α*, IL-1*β*, IL-2, IL-25, IL-3, IL-33, IL-4, IL-5, IL-6, IL-7, IL-8, IL-9, monocyte chemotactic protein (MCP)1, MCP-1, MCP-3, regulated upon activation normally T expressed and presumably secreted (RANTES), tumour necrosis factor-alpha (TNF-*α*), TNF-*β*, and thymic stromal lymphopoietin (TSLP). All detailed descriptions were displayed in Table S1. During data interpretation, cytokine values below the detection limit were imputed by utilizing robust regression on order statistics as previously described [18].

### 2.3. Validation Cohort and Potential Cytokine Validation

To further confirm the predictive values of potential biomarkers, another independent cohort consisting of 60 primary CRSwNP and 60 recurrent CRSwNP was conducted, and serum samples were collected. The levels of potential cytokines were detected with a commercial enzyme-linked immunosorbent assay (ELISA) kit (CUSABIO, Wuhan, China) according to the manufacturer's instructions.

### 2.4. Statistical Analysis

Numerical variable data were described by mean ± standard deviation, and Student's *t*-test was used when the variables were normally distributed, and the Mann–Whitney *U*-test was used when the data were not normally distributed. Categorical variable data were expressed as frequencies and percentages, and chi-square tests were used to compare differences. Receiver-operating characteristic (ROC) curves were constructed to explore potential cytokine and assess their value in predicting CRSwNP recurrence. All statistical analyses were performed on SPSS statistical software version 25.0 (IBM, Chicago, IL, USA). For all tests, *P* < 0.05 was considered to be statistically significant.

## 3. Results

### 3.1. Demographics and Clinical Data of Participants

A total of 72 patients were included in the discovered cohort, including 36 primary CRSwNP and 36 recurrent CRSwNP. The clinical data and demographics are shown in Table 1. Blood eosinophil count and percentage were significantly higher in the recurrent group than in the primary group (*P* < 0.05). No statistical difference was observed in age, gender, BMI, concomitant diseases, baseline VAS and Lund-Kennedy score, and Lund-Mackay score (all *P* > 0.05).

### 3.2. Different Clustering of Cytokines and Potential Predictive Value for Postoperative Recurrence

**Figure 1:**
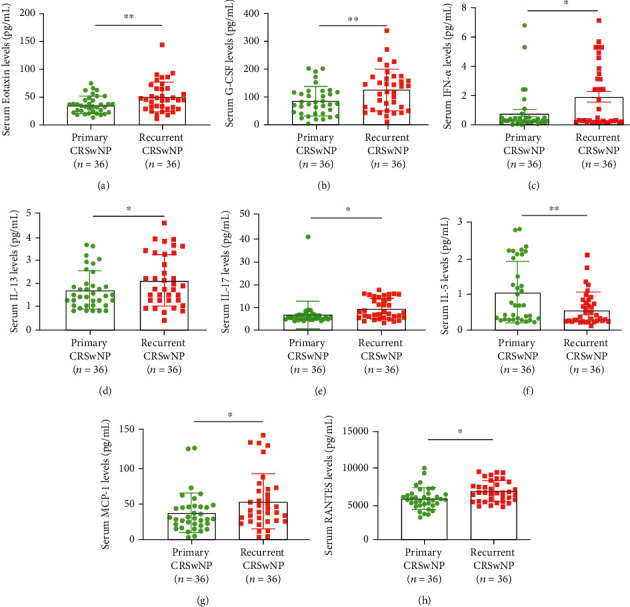
Serum levels of 8 cytokines were dysregulated between primary CRSwNP and recurrent CRSwNP groups based on multiple cytokine profiling. CRSwNP; chronic rhinosinusitis with nasal polyps; G-CSF: granulocyte colony-stimulating factor; IFN-*α*: interferon alpha; IL: interleukin; MCP: monocyte chemotactic protein; RANTES: regulated upon activation normally T expressed and presumably secreted. ^∗^*P* < 0.05, ^∗∗^*P* < 0.01.

**Figure 2:**
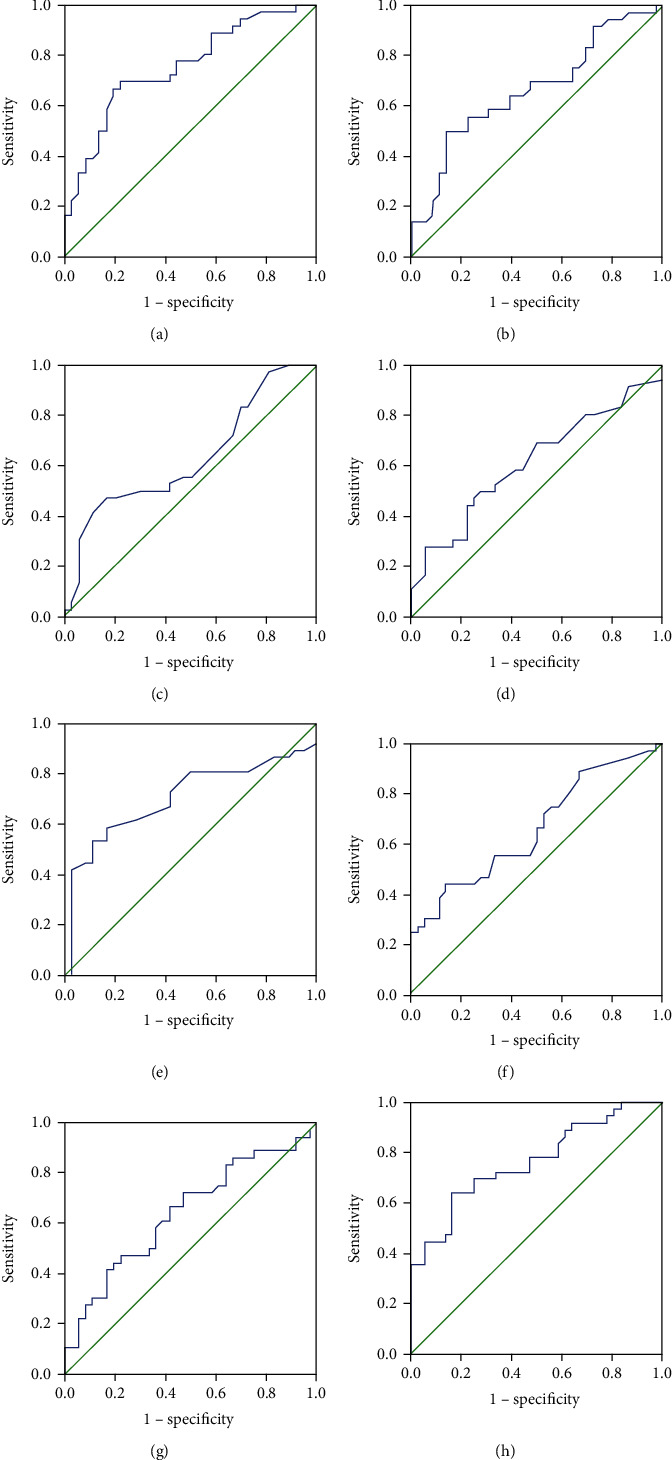
ROC curves of potential predictive cytokines for postoperative recurrence of CRSwNP. (a) eotaxin; (b) G-CSF; (c) IFN-*α*; (d) IL-13; (e) IL-17A; (f) IL-6; (g) MCP-1; (h) RANTES. ROC: receiver-operating characteristic; CRSwNP: chronic rhinosinusitis with nasal polyps; G-CSF: granulocyte colony-stimulating factor; IFN-*α*: interferon alpha; IL: interleukin; MCP: monocyte chemotactic protein; RANTES: regulated upon activation normally T expressed and presumably secreted.

In the present study, the levels of 30 cytokines were measured in the serum samples, and their abbreviations and descriptive statistics were exhibited in Table S1. As shown in Table 2 and Figure 1, serum levels of eotaxin, G-CSF, IFN-*α*, IL-13, IL-17A, IL-5, MCP-1, and RANTES were increased in the recurrent group, while IL-6 levels were decreased. ROC curves were performed, and the results indicated that eotaxin, G-CSF, IFN-*α*, IL-17A, IL-6, and MCP-1, and RANTES exhibited potential values in predicting the postoperative recurrence of CRSwNP, and eotaxin, IL-17A, and RANTES showed stronger predictive ability than other indicators (AUC > 7.0, *P* < 0.05) (Figure 2). Detailed parameters are displayed in Table 3.

### 3.3. Validation of Cytokines by ELISA

**Figure 3:**
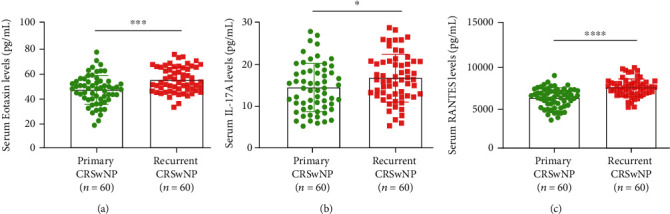
The serum levels of most potential cytokines between primary CRSwNP and recurrent CRSwNP groups in the validation cohort. (a) eotaxin; (b) IL-17; (c) RANTES. CRSwNP: chronic rhinosinusitis with nasal polyps; IL: interleukin; RANTES: regulated upon activation normally T expressed and presumably secreted. ^∗^*P* < 0.05, ^∗∗∗^*P* < 0.001.

**Figure 4:**
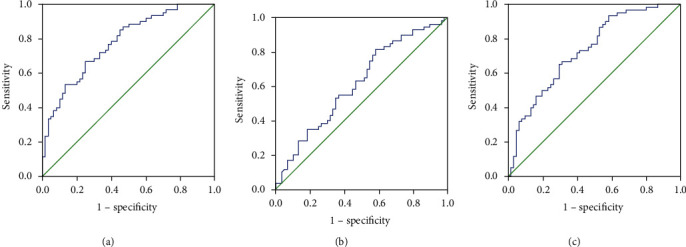
ROC curves of most potential cytokines for postoperative recurrence of CRSwNP. (a) Eotaxin; (b) IL-17; (c) RANTES. ROC: receiver-operating characteristic; CRSwNP: chronic rhinosinusitis with nasal polyps; IL: interleukin; RANTES: regulated upon activation normally T expressed and presumably secreted.

To further examine the reliability and reproducibility of results obtained in the discovery cohort, we conducted a validation cohort containing 60 primary patients and 60 recurrent patients. Concomitant AR rate, blood eosinophil count, and percentage were significantly elevated in the recurrent group than in the primary group (*P* < 0.05); no statistical difference was found in other baseline data between the two groups (all *P* > 0.05) (Table 4). The ELISA results demonstrated that serum eotaxin, IL-17A, and RANTES levels were significantly higher in the recurrent group than in the primary group (all *P* < 0.05) (Figure 3). Furthermore, the ROC curve in Figure 4 suggested that serum eotaxin (AUC = 0.729, *P* < 0.001) and RANTES (AUC = 0.776, *P* < 0.001) showed stronger ability than serum IL-17A (AUC = 0.617, *P* = 0.027) in predicting CRSwNP recurrence. Detailed data are presented in Table 5.

## 4. Discussion

CRSwNP is a heterogeneous nasal mucosal disease with a high prevalence worldwide [19, 20]. Although current FESS could markedly improve the clinical symptoms of patients, there remained a large proportion of patients who suffered a relapse. Therefore, an early and appropriate method to predict the prognosis and recurrence of CRSwNP are extremely important. Although several biomarkers, circulating inflammatory cells, or methods associated with CRSwNP recurrence have been described [21–25], these indicators need to be further validated because of their weak sensitivity and specificity. To address this issue, we performed this discover-validation study, combining the application of multiplex assays and further ELISA to explore potential predictive biomarkers for postoperative recurrence in patients with CRSwNP.

In the present study, our results demonstrated that 8 cytokine levels were significantly changed between recurrent CRSwNP groups, and serum eotaxin, IL-17A, and RANTES were proven to exhibit powerfully predictive abilities with high accuracy included. To further validate the reliability of the results, serum eotaxin, IL-17A, and RANTES levels were detected in a validation cohort of 120 patients. Finally, our study demonstrated that serum eotaxin and RANTES might be reliable biomarkers for predicting CRSwNP recurrence.

The chemokine ligands (CCL) of the eotaxin family consist of eotaxin-1 (CCL11), eotaxin-2 (CCL24), and eotaxin-3 (CCL26) were reported to be involved in immune regulation and inflammatory response [26]. Previous studies found that eotaxin could promote the activation, maturation, and migration of eosinophils, which contributed to the eosinophil infiltration in the pathological processes of various autoimmune and inflammatory diseases [27, 28]. Recent publications presented that serum eotaxin-3 levels were significantly elevated in allergic patients than in healthy controls [29, 30]. Moreover, eotaxin-3 was demonstrated to be involved in the pathogenesis of eosinophilic esophagitis and associated with disease prognosis [31–33]. Accordingly, Th2 cytokines significantly increased eotaxin-3 production by human nasal fibroblasts, and the increased eotaxin-3 level in tissues could induce eosinophil infiltration in mucosa resulting in eosinophilic inflammation [28]. In this study, we found that eotaxin levels were overexpressed in the serum of patients with recurrent CRSwNP, suggesting that serum eotaxin levels might be associated with postoperative recurrence. Interestingly, we identified another CCL member, RANTES, which was also notably elevated in the serum of recurrent patients. RANTES, also known as CCL5, was expressed on various cell types, including fibroblasts, epithelial cells, and eosinophil, and exhibits multiple biological functions, such as angiogenesis, tissue remodelling, antigen presentation, chronic inflammation, and fibrosis by binding to its receptor [34]. Several studies described that RANTES was a key chemokine which was involved in the recruitment of eosinophils during allergic airway inflammation, and it was regarded as a pivotal regulator in eosinophilic disease driven by Th2 cells [35, 36]. It is well known that CRSwNP is a chronic inflammatory disease predominately characterized by Th2 inflammation and eosinophilic inflammation [5, 37]. Previous publications showed that CRSwNP patients with excessive eosinophil counts and Th2 cytokine levels in polyps specimens were more likely to experience postoperative relapse [38]. Combined with our results, we hypothesized that high levels of circulating eotaxin and RANTES could promote eosinophil infiltration into nasal polyps tissues, then increase the risk of postoperative recurrence in CRSwNP patients. However, the underlying mechanisms are not clear and need further identification.

Another important finding was that serum IL-17A concentrations produced by Th17 cells were significantly higher in the recurrent CRSwNP group than in the primary CRSwNP group. IL-17A was proved to be involved in airway inflammation and airway remodelling in airway diseases, and its levels positively correlated with the severity of asthma [39, 40]. Moreover, recent studies reported that IL-17A played a critical role in eosinophil aggregation and tissue remodelling in CRSwNP [41]. Prior studies found that the imbalance of Th17/Treg cells played a vital role in the occurrence and development of CRSwNP [42–44]. Furthermore, prior publications found that Th17 cells were involved in the induction of immune-related tissue damage, which contributed to the pathophysiology of inflammatory diseases, including systemic lupus erythematosus, asthma, and CRSwNP [44–47]. In the present study, we found that serum IL-17A levels were elevated in recurrent patients in both discovery and validation cohorts, and serum IL-17A exhibits a potential predictive value for CRSwNP recurrence. Therefore, we assumed that high levels of serum IL-17A reflected the degree of Th17 inflammation and contributed to the accumulation of eosinophils in the tissues and tissue remodelling, leading to an increased risk of postoperative recurrence in CRSwNP patients.

Our study has several limitations. First, the conclusion in this study was limited because of relatively small sample size in a single medical centre. Second, other cytokines with low AUC values were not validated. It did not mean that they exhibited poor predictive values. Finally, we did not evaluate the multiple cytokine profiling between CRSwNP and healthy controls.

## 5. Conclusion

This was the first application of multiple cytokine analysis to explore potential biomarkers for predicting postoperative recurrence of CRSwNP. We found that serum eotaxin, RANTES, and IL-17A might serve as powerful biomarkers for predicting postoperative recurrence in CRSwNP patients. These results strengthened the evidence that cytokine levels were associated with CRSwNP recurrence and contributed to the understanding of potential mechanisms of postoperative recurrence. Further prospective multicenter studies with larger sample sizes will allow us to draw more definitive conclusions about the efficacy of serum cytokines in predicting CRSwNP recurrence.

## Figures and Tables

**Table 1:** The demographic and clinical variables between the two groups.

Variables	Primary CRSwNP (*n* = 36)	Recurrent CRSwNP (*n* = 36)	*P* value
Age, years	41.0 ± 10.9	40.4 ± 12.1	0.831
Gender (male/female)	21/15	18/18	0.637
BMI, kg/m^2^	23.4 ± 1.8	23.2 ± 1.9	0.750
AR (yes/no)	5/31	11/25	0.155
AS (yes/no)	3/33	7/29	0.307
Blood eosinophil count, 10^9^/L	0.2 ± 0.1	0.3 ± 0.1	0.015
Blood eosinophil percentage, %	3.6 ± 1.6	5.1 ± 2.1	<0.001
VAS score	5.2 ± 1.7	5.7 ± 1.8	0.180
Lund-Kennedy score	8.9 ± 2.1	8.5 ± 1.8	0.473
Lund-Mackay score	17.8 ± 1.8	18.7 ± 2.3	0.072

CRSwNP: chronic rhinosinusitis with nasal polyps; BMI: body mass index; AR: allergic rhinitis; AS: asthma; VAS: visual analogue scale.

**Table 2:** The comparisons of cytokine levels between two groups.

Cytokines	Primary CRSwNP (*n* = 36)	Recurrent CRSwNP (*n* = 36)	*P* value
CTACK	1002.9 ± 311.3	1047.1 ± 380.6	0.591
Eotaxin	35.8 ± 15.2	50.7 ± 27.2	0.005
G-CSF	86.8 ± 52.6	127.9 ± 74.6	0.009
GM-CSF	1.0 ± 0.7	1.1 ± 0.8	0.296
IFN-*α*	0.7 ± 1.4	1.9 ± 2.1	0.010
IFN-*γ*	6.4 ± 3.3	5.3 ± 2.4	0.092
IL-10	3.0 ± 4.4	2.7 ± 5.5	0.791
IL-13	1.7 ± 0.8	2.2 ± 1.0	0.039
IL-15	37.8 ± 16.8	37.5 ± 20.5	0.950
IL-16	104.8 ± 62.9	123.8 ± 63.8	0.207
IL-17A	6.7 ± 5.9	9.3 ± 4.5	0.042
IL-18	34.1 ± 23.7	36.6 ± 17.8	0.612
IL-1*α*	10.1 ± 7.3	10.8 ± 5.9	0.665
IL-1*β*	2.2 ± 1.4	2.6 ± 0.9	0.097
IL-2	0.5 ± 0.3	0.6 ± 0.3	0.489
IL-25	482.0 ± 580.6	480.9 ± 363.3	0.992
IL-3	11.2 ± 18.7	11.6 ± 8.6	0.918
IL-33	222.5 ± 23.6	221.0 ± 23.3	0.778
IL-4	1.9 ± 0.5	1.9 ± 0.6	0.630
IL-5	2.4 ± 2.3	2.6 ± 2.7	0.718
IL-6	1.0 ± 0.9	0.6 ± 0.5	0.005
IL-7	5.6 ± 5.4	6.2 ± 5.6	0.616
IL-8	105.1 ± 72.5	96.3 ± 64.1	0.589
IL-9	233.4 ± 22.4	236.4 ± 25.2	0.603
MCP-1	36.2 ± 26.6	51.8 ± 36.9	0.044
MCP-3	1.6 ± 1.9	2.1 ± 2.6	0.345
RANTES	5902.4 ± 1400.5	6518.6 ± 1571.1	0.038
TNF-*α*	18.4 ± 6.1	18.5 ± 7.3	0.928
TNF-*β*	74.9 ± 20.4	76.8 ± 24.4	0.717
TSLP	841.9 ± 394.1	957.4 ± 354.8	0.196

CRSwNP: chronic rhinosinusitis with nasal polyps; CTACK: cutaneous T cell attracting chemokine; G-CSF: granulocyte colony-stimulating factor; GM-CSF: granulocyte-macrophage colony-stimulating factor; IFN: interferon alpha; IL: interleukin; MCP: monocyte chemotactic protein; RANTES: regulated upon activation normally T expressed and presumably secreted; TNF: tumour necrosis factor-alpha; TSLP: thymic stromal lymphopoietin.

**Table 3:** ROC analysis results of different cytokines for predicting CRSwNP recurrence.

Cytokines	AUC (95% CI)	*P* value	Cutoff value	Sensitivity	Specificity
Eotaxin	0.753 (0.642-0.865)	<0.001	43.6	0.667	0.806
G-CSF	0.665 (0.539-0.791)	0.016	134.0	0.500	0.861
IFN-*α*	0.636 (0.506-0.766)	0.047	1.4	0.444	0.861
IL-13	0.615 (0.484-0.746)	0.092	3.2	0.278	0.944
IL-17A	0.703 (0.576-0.830)	0.003	9.8	0.417	0.972
IL-6	0.666 (0.541-0.790)	0.016	1.0	0.44	0.861
MCP-1	0.642 (0.514-0.771)	0.038	47.6	0.417	0.833
RANTES	0.764 (0.654-0.873)	<0.001	6426.0	0.639	0.831

ROC: receiver-operating characteristic; CRSwNP: chronic rhinosinusitis with nasal polyps; AUC: area under the curve; CI: confidence interval; G-CSF: granulocyte colony-stimulating factor; IFN-*α*: interferon alpha; IL: interleukin; MCP: monocyte chemotactic protein; RANTES: regulated upon activation normally T expressed and presumably secreted.

**Table 4:** The demographic and clinical variables between the two groups.

Variables	Primary CRSwNP (*n* = 60)	Recurrent CRSwNP (*n* = 60)	*P* value
Age, years	39.7 ± 9.1	41.0 ± 10.1	0.831
Gender (male/female)	35/25	28/32	0.273
BMI, kg/m^2^	22.8 ± 1.8	22.6 ± 1.4	0.637
AR (yes/no)	9/51	21/39	0.019
AS (yes/no)	7/53	13/47	0.220
Blood eosinophil count, 10^9^/L	0.2 ± 0.1	0.3 ± 0.1	<0.001
Blood eosinophil percentage, %	3.4 ± 1.5	4.9 ± 2.2	<0.001
VAS score	5.3 ± 1.7	6.0 ± 2.2	0.061
Lund-Kennedy score	9.5 ± 2.1	9.5 ± 2.2	0.966
Lund-Mackay score	18.3 ± 3.3	17.5 ± 2.9	0.125

CRSwNP: chronic rhinosinusitis with nasal polyps; BMI: body mass index; AR: allergic rhinitis; AS: asthma; VAS: visual analogue scale.

**Table 5:** ROC analysis results of different cytokines for predicting CRSwNP recurrence.

Cytokines	AUC (95% CI)	*P* value	Cutoff value	Sensitivity	Specificity
Eotaxin	0.729 (0.640-0.818)	<0.001	50.3	0.650	0.700
IL-17A	0.617 (0.516-0.717)	0.027	12.0	0.817	0.417
RANTES	0.776 (0.695-0.858)	<0.001	7177.7	0.667	0.750

ROC: receiver-operating characteristic; CRSwNP: chronic rhinosinusitis with nasal polyps; AUC: area under the curve; CI: confidence interval; IL: interleukin; RANTES: regulated upon activation normally T expressed and presumably secreted.

## Data Availability

The data used to support the observations of this study are available from the corresponding author upon request.

## Supplementary Materials

Supplementary Materials Table S1: 1, circulating 30 cytokines, abbreviations, and statistical descriptions (pg/mL).
