# Supplementary material for: Exploration of Predictive Biomarkers for Postoperative Recurrence in Chronic Rhinosinusitis with Nasal Polyps Based on Serum Multiple-Cytokine Profiling
Source: Mediators Inflamm. 2022 Sep 28;2022:1061658. doi: 10.1155/2022/1061658 (PMC9534722; doi:10.1155/2022/1061658)
Supplement: Supplementary Materials — Table S1: 1, circulating 30 cytokines, abbreviations, and statistical descriptions (pg/mL). [file 1061658.f1.docx]

| Table S1 Circulating 30 cytokines, abbreviations, and statistical descriptions (pg/ml) | | | |
| --- | --- | --- | --- |
| Cytokines | Abbreviation | Mean (± SD) | Range |
| Cutaneous T cell attracting chemokine | CTACK | 1025.1 ± 345.9 | 288.7-2156.0 |
| Eotaxin | Eotaxin | 43.6 ± 23.0 | 12.3-141.0 |
| Granulocyte colony stimulating factor | G-CSF | 107.3 ± 67.3 | 5.7-340.4 |
| Granulocyte-macrophage colony stimulating factor | GM-CSF | 1.0 ± 0.8 | 0.1-3.4 |
| Interferon alpha | IFN-α | 1.3 ± 1.9 | 0-7.2 |
| Interferon gamma | IFN-γ | 5.9 ± 2.8 | 2.0-13.3 |
| Interleukin-10 | IL-10 | 2.9 ± 4.9 | 0.3-34.2 |
| Interleukin-13 | IL-13 | 1.9 ± 1.0 | 0.4-4.6 |
| Interleukin-15 | IL-15 | 37.6 ± 18.6 | 11.3-96.9 |
| Interleukin-16 | IL-16 | 114.3 ± 63.6 | 18.7-363.1 |
| Interleukin-17A | IL-17A | 8.0 ± 5.4 | 3.3-41.7 |
| Interleukin-18 | IL-18 | 35.3 ± 20.9 | 0.9-132.3 |
| Interleukin-1 alpha | IL-1α | 10.4 ± 6.6 | 1.5-34.3 |
| Interleukin-1beta | IL-1β | 2.4 ± 1.3 | 0.6-9.9 |
| Interleukin-2 | IL-2 | 0.6 ± 0.3 | 0.1-1.6 |
| Interleukin-25 | IL-25 | 481.5 ± 480.9 | 104.9-3358.0 |
| Interleukin-3 | IL-3 | 11.4 ± 14.4 | 2.5-96.9 |
| Interleukin-33 | IL-33 | 221.7 ± 23.3 | 156.4-263.1 |
| Interleukin-4 | IL-4 | 1.9 ± 0.6 | 1.0-3.6 |
| Interleukin-5 | IL-5 | 2.5 ± 2.5 | 0.4-13.7 |
| Interleukin-6 | IL-6 | 0.8 ± 0.7 | 0.1-2.8 |
| Interleukin-7 | IL-7 | 5.8 ± 5.9 | 0.9-29.6 |
| Interleukin-8 | IL-8 | 100.7 ± 68.1 | 4.7-293.2 |
| Interleukin-9 | IL-9 | 234.9± 23.7 | 163.8-306.3 |
| Monocyte chemotactic protein 1 | MCP-1 | 44.0 ± 32.9 | 5.6-140.4 |
| Monocyte chemotactic protein 3 | MCP-3 | 1.8 ± 2.3 | 0.1-8.9 |
| Regulated upon activation normally T expressed and presumably secreted | RANTES | 6210.5 ± 1501.0 | 3326.5-9931.0 |
| Tumor necrosis factor-alpha | TNF-α | 18.5 ± 6.7 | 10.1-42.7 |
| Tumor necrosis factor-beta | TNF-β | 75.8 ± 22.4 | 37.0-141.5 |
| Thymic stromal lymphopoietin | TSLP | 899.6 ±376.6 | 213.7-2049.0 |

SD, standard deviation.
